# KARAOKE: Krill oil versus placebo in the treatment of knee osteoarthritis: protocol for a randomised controlled trial

**DOI:** 10.1186/s13063-019-3915-1

**Published:** 2020-01-14

**Authors:** L. L. Laslett, B. Antony, A. E. Wluka, C. Hill, L. March, H. I. Keen, P. Otahal, F. M. Cicuttini, G. Jones

**Affiliations:** 1 Menzies Institute for Medical Research, University of Tasmania, Private Bag 23, Hobart, TAS 7000 Australia; 2 Department of Epidemiology and Preventive Medicine, Monash University, Alfred Hospital, Melbourne, VIC 3004 Australia; 3 The Queen Elizabeth Hospital, University of Adelaide, Woodville, SA 5011 Australia; 4 Discipline of Medicine, University of Adelaide, Adelaide, SA 5005 Australia; 5 Royal North Shore Hospital, The University of Sydney, Sydney, NSW 2006 Australia; 6 Department of Rheumatology, Fiona Stanley Hospital, Murdoch Drive, Murdoch, WA 6150 Australia; 7 Medical School, University of Western Australia, Crawley, 6009 WA Australia

**Keywords:** Krill oil, Osteoarthritis, Pain, Knee pain, Magnetic resonance imaging (MRI)

## Abstract

**Background:**

Knee osteoarthritis (OA) is a common and important cause of pain and disability, but interventions aimed at modifying structures visible on imaging have been disappointing. While OA affects the whole joint, synovitis and effusion have been recognised as having a role in the pathogenesis of OA. Krill oil reduces knee pain and systemic inflammation and could be used for targeting inflammatory mechanisms of OA.

**Methods/design:**

We will recruit 260 patients with clinical knee OA, significant knee pain and effusion-synovitis present on MRI in five Australian cities (Hobart, Melbourne, Sydney, Adelaide and Perth). These patients will be randomly allocated to the two arms of the study, receiving 2 g/day krill oil or inert placebo daily for 6 months. MRI of the study knee will be performed at screening and after 6 months. Knee symptoms, function and MRI structural abnormalities will be assessed using validated methods. Safety data will be recorded. Primary outcomes are absolute change in knee pain (assessed by visual analog score) and change in size of knee effusion-synovitis over 24 weeks. Secondary outcomes include improvement in knee pain over 4, 8, 12, 16 and 20 weeks. The primary analyses will be intention-to-treat analyses of primary and secondary outcomes. Per protocol analyses adjusting for missing data and for treatment compliance will be performed as the secondary analyses.

**Discussion:**

This study will provide high-quality evidence to assess whether krill oil 2 g/day reduces pain and effusion-synovitis size in older adults with clinical knee OA and knee effusion-synovitis. If krill oil is effective and confirmed to be safe, we will provide compelling evidence that krill oil improves pain and function, changes disease trajectory and slows disease progression in OA. Given the lack of *approved therapies* for slowing disease progression in OA, and moderate cost of krill oil, these findings will be readily translated into clinical practice.

**Trial registration:**

Australian New Zealand Clinical Trials Registry, ACTRN12616000726459. Registered on 02 June 2016.

Universal Trial Number (UTN) U1111–1181-7087.

## Background

Osteoarthritis (OA) is a major cause of pain, functional limitation and disability worldwide [1], with hip and knee OA ranked as the 11th highest contributor to global disability and 38th highest in disability-adjusted life years (DALYs) [2]. Treatment remains focussed on managing pain, which is ranked by patients as the highest treatment priority [3]. However, pain control remains poor in 50% of non-operatively treated patients [4]. Despite the large disease burden, currently no approved disease modifying OA drugs (DMOADs) are available.

OA is a heterogeneous, complex disease with multiple phenotypes [5, 6]. Few treatments have been demonstrated to be effective for OA pain and to slow down changes in structure, and this may be partly due to treating everyone as if they have the same pathological process. Treatment can be optimised by selecting study populations by subgroups with specific features that are likely to respond to targeted treatments. One such phenotype is an inflammatory phenotype.

OA is typically considered a ‘non-inflammatory’ type of arthritis; however, localised low-grade inflammation is an important factor in OA pathogenesis, with inflammatory patterns observed both early [7] and late [8] in the disease process. In OA, inflammatory changes have been demonstrated both systemically and in the affected joint. Moreover, there is now evidence that inflammation helps distinguish clinically distinct phenotypes of OA [9].

Elevated levels of systemic inflammation (as observed by high sensitive C reactive protein (hsCRP) [10] are observed in persons with OA compared to controls, and are positively correlated with the degree of synovial inflammatory infiltration in OA [11]. hsCRP levels are associated with both symptoms and disease markers, with higher hsCRP levels associated with greater pain (in both a meta-analysis [10] and longitudinal data from our centre [12]), decreased physical function [10], decreased cartilage volume [13] and disease progression [7, 14]. Therefore, systemic inflammation predicts both pain and structural outcomes in OA.

Inflammation also occurs locally within joints. Pro-inflammatory cytokines, including interleukin (IL)-1β, tumour necrosis factor-α (TNF-α), and IL-6 are produced by synovium and chondrocytes, and contribute to the progression of cartilage degradation [8]. Localised inflammation presents as effusion (excess synovial fluid within the joint space) and/or synovitis (thickening of the synovium); these predict pain [15–18], including new and worsening pain over 2.6 years [18]. Effusion-synovitis also predicts structural changes— cartilage defects, bone marrow lesions (BMLs) and increased cartilage loss over 2.6 years [19]—and joint replacement [20]. Thus, stopping the cascade of inflammation is likely to slow down deleterious changes in knee structure and reversing inflammation has the potential to improve outcomes globally in knee OA.

High levels of inflammation in OA can be targeted with treatments including oral prednisolone [21] or biologics [22], but these therapies are expensive and have too many side effects for widespread use. Safer treatments for reducing inflammation are needed.

Fish oil is effective in people with rheumatoid arthritis (RA), reducing pain [23], morning stiffness and number of painful and/or tender joints [23, 24], and non-steroidal anti-inflammatory drug (NSAID) consumption [23, 25]. This is an effective add-on therapy to standard RA therapies, reducing the risk of treatment failure and increasing the rate of remission [26]. Efficacy data on the use of marine-sourced oils is more limited in people with OA. Observational data suggest that omega-6 and omega3 polyunsaturated fatty acids from dietary sources may have beneficial effects on synovitis and cartilage damage cross-sectionally [27], but that use of a variety of marine oils is ineffective for OA pain [28, 29], although there is marked heterogeneity amongst the predominantly poor quality trials [28], and none were enriched for participants with evidence of an inflammatory phenotype. Canola oil/low dose fish oil may be effective [29]. However, unlike marine oils in general, oil from Antarctic krill (*Ephausia superba*, a zooplankton crustacean) may be effective in people with OA. Like fish oil, it is high in eicosapentanoic acids (EPA) and decosahexanoic acid (DHA) [30], although the chemical structures of the fatty acids differ (phospholipids, rather than triacylglycerol or fatty acid ethyl esters); but unlike fish oil, it also naturally contains antioxidants (predominantly astaxanthin [30]). Additionally, bioavailability of krill oil is better than fish oil, as comparable amounts of EPA and DHA are obtained from lower doses of krill oil compared to fish oil [31]. In animal studies, krill oil supplementation had larger effects on most clinical outcomes than fish oil [32], while krill oil reduced the severity of inflammatory arthritis in mice by 50% compared to controls [32]. In mice transgenic for human TNF-α, both fish and krill oil improved plasma cholesterol levels, but only krill oil had additional beneficial effects on markers of fatty acid oxidation [33]. Therefore, bioavailability data and animal studies suggest that krill oil has anti-inflammatory and anti-oxidative effects and may be a better treatment in vivo than fish oil.

Two randomised controlled trials (RCTs) including people with osteoarthritis have demonstrated that daily krill oil (300 mg [34] or 2 g [35]) is effective in reducing some aspect of knee pain [34, 35], functional impairment [34, 35], and stiffness [35]. However, these RCTs have methodological limitations, including being of short duration (30 days), having poor documentation of adverse events, not including any imaging data to determine the effect of krill oil on knee structures, and not targeting patients with evidence of inflammation.

Therefore, we aim to compare, using a randomised, placebo-controlled double-blind design over 6 months, the effect of 2 g krill oil daily compared to identical placebo, on knee pain and MRI-detected knee effusion-synovitis size (primary outcomes) in participants with clinical knee OA, significant knee pain and effusion-synovitis on MR imaging over 24 weeks.

## Objective

We are conducting a multi-centre randomised, placebo-controlled double-blind clinical trial. This will compare efficacy of krill oil vs identical placebo to treat knee OA (both pain and structure) in 260 patients with clinical knee OA, significant knee pain and effusion-synovitis on imaging.

We hypothesise that krill oil (2 g daily) will decrease pain (assessed by 100 mm visual analog scale (VAS)) score by 10 mm more than identical placebo over 24 weeks and decrease effusion-synovitis size over 24 weeks (co-primary hypotheses) and improve knee pain over 4, 8, 12, 16, and 20 weeks (secondary hypotheses) in patients with symptomatic knee OA and knee effusion-synovitis, compared with placebo. If krill oil proves effective, it will offer a novel therapeutic approach to reduce knee OA progression.

## Methods/design

### Study design

This randomised trial of krill oil for OA of the knee (KARAOKE) study is a multicentre, randomised, double-blind, placebo-controlled superiority trial over 24 weeks. The trial was registered on the Australian New Zealand Clinical Trials Registry prior to recruitment, and trial reporting will be guided by the Consolidated Standards of Reporting Trials (CONSORT) statement [36]. We aim to recruit a convenience sample of 260 patients with clinical knee OA, significant knee pain and effusion-synovitis visualised on MRI. Patients will be recruited via the OA Clinical Trial Network, at Australian public hospitals in Melbourne, Sydney, Adelaide and Perth and a research institute in Hobart, using a combined strategy, including collaboration with general practitioners, rheumatologists and orthopaedic surgeons, as well as advertising through local and social media. Patients will be encouraged to contact their local research nurse via email or telephone.

### Inclusion criteria

Inclusion criteria are as follows: males and females aged ≥ 40 years; with significant knee pain on most days (defined as a pain score ≥ 40 mm on a 100-mm VAS); and meeting the American College of Rheumatology (ACR) criteria for symptomatic knee OA [37], assessed by a physician, and any effusion-synovitis present on MRI (defined as grade 1 or more according to modified WORMS scoring [18, 38]).

### Exclusion criteria

Exclusion criteria are as follows:

1. Significant knee injury within the last 6 months
2. Use of anticoagulants, high dose aspirin or NSAIDs, as krill oil is contraindicated in such people
3. Unwillingness to stop taking krill oil and fish oil medications 30 days prior to the trial and during the trial (minimum washout 4 weeks)
4. Other forms of inflammatory arthritis (especially rheumatoid arthritis and gout)
5. Seafood allergy
6. Arthroscopy or open surgery in the “study” knee in the last 12 months
7. Injections of corticosteroids (last 3 months) or hyaluronic acid (last 6 months) in the index knee
8. Women who are pregnant or breastfeeding
9. Use of any investigational drug(s) and/or devices within 30 days prior to randomisation
10. Presence of any serious medical illness that may preclude 24-week follow-up
11. Inability to provide informed consent
12. Inability to have an MRI (claustrophobia, pacemakers, metal in eyes, metal in knees that disrupt the images at the area of interest)
13. Severe knee OA (joint space narrowing (JSN)) on x-ray of grade 3 using the Osteoarthritis Research Society International (OARSI) atlas [39])

### Randomisation and blinding

Study participants were allocated to receive either krill oil or placebo in a 1:1 ratio based on computer-generated random numbers using a central randomisation website hosted by the University of Tasmania using adaptive allocation (minimisation) [40]. Briefly, the randomisation program examined the number of participants that were currently assigned to each of the two arms and then adjusted the randomisation thresholds so that the arm with the fewest participants would have a greater chance of being selected. Randomisation will be stratified by study site and is accessible by a dedicated website. This will be conducted by staff members with no direct involvement in the study.

The randomised controlled trial will be a double-blind one, with both patients and investigators assessing outcomes blinded to treatment allocation. Allocation concealment and double blinding will be ensured by: 1) use of identical softgels for each group; 2) objective measures of knee structural changes being made by trained observers blinded to group allocation; and 3) subjective measures being taken by research nurses blinded to group allocation.

Emergency unblinding will be allowed in limited situations that impact on the safety of study patients. Code-break for the full randomisation schedule will be maintained by the University of Tasmania. Patients who are unblinded will be withdrawn from treatment but will continue to be followed as per the planned follow-up schedule.

### Intervention

Eligible people will receive 2 × 1 g softgels daily of either krill oil or placebo for 6 months. The krill oil (Superba Boost product) contains 190 mg/g of EPA and 100 mg/g of DHA. The total omega-3 content is 350 mg/g, total omega-6 is 12 mg/g, hence the omega-3 to omega-6 ratio is 29. The placebo used is a mixture of vegetable oils (virgin cold pressed olive oil, maize oil, palm kernel oil, medium chain triglycerides), containing no EPA or DHA, and less than 5 mg/g (0.5%) other omega-3s (predominantly oleic acid (C18:1n9) and linoleic acid (C18:2n6)). Both the krill oil and the placebo are provided in non-distinguishable opaque glycerin softgels. A small amount of vanilla flavour added to the shell of both active and placebo softgels to ensure uniform taste and smell. All patients will continue usual care by their treating health practitioners.

### Study procedure and time points

Research assistants will first conduct screening over the telephone. If early checks of study eligibility are favourable, participants will be booked in for a face-to-face screening visit to further determine eligibility and explain what is involved in the study. At the screening face-to-face visit, patients will complete questionnaires, have a knee x-ray and MRI, supply a blood sample and have a clinical assessment by a study doctor to ensure inclusion criteria are met. The study knee will be defined as the one with symptomatic OA meeting all inclusion criteria. If both knees meet these criteria, the study doctor will decide which is the study knee, typically the one with the highest pain score.

Table 1 outlines the schedule of assessments. After screening, there will be three study visits (weeks 0, 12 and 24). The same research assistants, who are blinded to treatment allocation, will measure all clinical variables, administer questionnaires, monitor compliance and record adverse events at these visits. Additional questionnaire mail outs will occur monthly. MRI scans will occur at screening and week 24; knee x-ray will be performed at screening; blood samples are taken at screening, 12 and 24 weeks, and urine samples are taken at baseline and week 24.

**Table 1:** Schedule of assessments, KARAOKE study

Visit/week number	Screening	Baseline (week 0)	4	8	12	16	20	24
Informed consent	x							
Knee x-ray	x							
Knee MRI	x*							x
Clinical measures								
Bloods	x				x			x
Clinical examination	x							
Leg strength		x			x			x
Height and weight		x						x
Capsules given		x			x			
Capsule count					x			x
Pressure pain testing (Melbourne/Perth only)		x			x			x
Ultrasound (Perth)		x						x
Questionnaire measures								
Knee VAS	x	x	x	x	x	x	x	x
Knee WOMAC		x	x	x	x	x	x	x
Knee ICOAP		x	x	x	x	x	x	x
Patient global evaluation		x	x	x	x	x	x	x
Joint replacement surgery	x				x			x
Concomitant medications	x				x			x
Safety (AEs)					x			x
Hand VAS, back VAS		x	x	x	x	x	x	x
AQoL		x			x			x
Patient health questionnaire (PHQ-9)		x						
Pain at other sites		x			x			x
Treatment guessing					x			x
painDETECT		x						
Adverse events	As required							
Early withdrawal	As required							

*if otherwise suitable

Participants will discontinue involvement in the study if they need to use drugs that are contraindicated (marine oils, anticoagulants, high dose aspirin, NSAIDs), they stop taking study medication or the participant or the site investigators request discontinuation.

### Quality assurance

To ensure high-quality execution of the trial in accordance with the protocol, all trial staff will be trained by the chief investigators and provided with a standard protocol book which contains details of standard operating procedures, trial contacts, visits, measurements, monitoring and case report forms. Data are collected using the same forms across sites, these data are checked by staff in Hobart and any discrepancies are clarified.

### Primary outcomes

We have two primary outcomes for this study, assessing improvements in symptoms and structural progression. These are change in knee pain assessed by VAS over 24 weeks and change in effusion-synovitis volume assessed from MRI. All outcomes and time points of assessment are listed in Table 2.

**Table 2:** Primary and secondary outcomes

Outcome	Time points
Primary outcomes	
Knee pain: VAS score	24 weeks
Knee effusion-synovitis: maximal area, volume, ordinal measures (MRI)	24 weeks
Secondary outcomes	
BML: maximal area	24 weeks
Effusion volume (ultrasound)^γ^	24 weeks
Knee pain: VAS score	4, 8, 12, 16, 20 weeks
Knee pain: total WOMAC pain, weight bearing and non-weight bearing pain	all time points
Hand and back pain (VAS)	All time points
Knee function	All time points
OMERACT–OARSI responder criteria [41]	All time points
hs-CRP	12 and 24 weeks
Blood lipids	12 and 24 weeks
Blood glucose	12 and 24 weeks
Leg strength	12 and 24 weeks
Analgesic use	All time points
Quality of life (AQoL-6D)	12 and 24 weeks
Pressure pain threshold testing on study knee^¥^	24 weeks
Adverse events	12 and 24 weeks
Medication persistence	12 and 24 weeks
Cost data: hospitalisation, medication use, joint replacement	

^γ^ Perth only

^¥^ Melbourne and Perth ONLY

### Other outcome measures

#### Pain intensity

Knee pain, back pain and hand pain will be assessed using a 100 mm VAS by asking “on this line, thinking about your study knee/back/most painful hand, where would you rate your pain, using the last 7 days as a time frame” over 4, 8, 12, 16 and 20 (but not 24) weeks.

We will also assess pain using the Western Ontario and McMasters Universities Osteoarthritis Index (WOMAC) [42], also over the preceding 7 days. Five items of the WOMAC pain scale in 100-mm VAS format [43] will be used to assess pain during walking on a flat surface, going up and down stairs, at night while in bed, sitting or lying, and standing upright during the last 7 days. Items will be summed to create a total WOMAC pain score (range 0–500). Incomplete items will be addressed according to the WOMAC user guide [44]. If only one item is missing, the remaining four items will be averaged and then multiplied by five. The WOMAC pain score will be considered invalid if more than one item is missing.

#### Knee function

Knee function will be assessed using WOMAC [42]. Seventeen items of the WOMAC function scale in 100-mm VAS format [43] will be used to assess function during descending stairs, ascending stairs, rising from sitting, standing, bending to floor/picking up an object, walking on flat surface, getting in/out of the car, going shopping, putting on socks/stockings, rising from bed, taking off socks/stockings, lying in bed, getting in/out of the bath, sitting, getting on/off the toilet, heavy domestic duties, and light domestic duties during the last 7 days. Items will be summed to create a total WOMAC function score (range 0–1700). If two or fewer items are missing, the remaining items will be averaged and then multiplied by 17 [44]. The WOMAC function score will be considered invalid if more than two items are missing.

#### Magnetic resonance imaging outcomes

An MRI scan of the “study” knee will be performed (screening, week 24). Knees will be imaged in the sagittal plane on a 1.5 T or 3 T whole-body magnetic resonance unit using a dedicated knee coil. Sequences will include T2-weighted fat saturation three-dimensional (3-D) fast spin echo sequence (effusion-synovitis volume, cartilage defects, BMLs); T1-weighted fat saturation 3-D gradient-recalled acquisition sequence (cartilage volume, cartilage thickness); and T2 mapping (Table 3).

**Table 3:** Magnetic resonance imaging sequences and parameters

Study site	Machine and coils	T2 weighted sagittal 3D	T1-weighted sagittal 3D	T2 mapping
Hobart	1.5 T whole-body MR unit (GE Optima 450 W, Milwaukee, USA), using a dedicated Transmit/Receive 8-channel knee coil where patient size permits, if body habitus is too large we use a 16 channel large GEM Flex coil	T2 weighted fat-saturated 3D fast spin echo sequence; repetition time 2300 ms; echo time 80 ms; field of view 18 cm; 256 × 256 matrix with interpolation Recon Voxel 0.35 × 0.35 × 1 mm; 2 excitation; slice thickness 1 mm	T1-weighted fat-saturated 3D gradient-recalled acquisition; flip angle 30 degrees; repetition time 38 ms; echo time 3 ms; field of view 16 cm; 256 × 160 matrix Reconstructed Voxel 0.625 × 1 × 1.5 mm; 1 excitation; slice thickness 1.5 mm	T2 mapping; repetition time 1100 ms; echo time 6.6 ms; field of view 16 cm; 320 × 224 matrix Reconstructed Voxel 0.5 × 0.714 × 3 mm; 1 excitation; slice thickness 3 mm
Perth	1.5 T whole-body MR unit (Siemens, Magnetom Avanto Fit, Erlangen, Germany), using a dedicated Transmit/Receive 15 channel knee coil	T2 weighted fat-saturated 3D fast spin echo sequence; flip angle mode T2; repetition time 1400 ms; echo time 89 ms; field of view 19 cm; 256 × 256 matrix with interpolation Recon Voxel 0.4 × 0.4 × 1.5 mm; 1 excitation; slice thickness 1.5 mm	T1-weighted fat-saturated 3D gradient-recalled acquisition; flip angle 10 degrees; repetition time 13.9 ms; echo time 6.05 ms; field of view 19 cm; 320 × 298 matrix Reconstructed Voxel 0.6 × 0.6 × 1.5 mm; 1 excitation; slice thickness 1.5 mm	T2 mapping (multi echo SE); flip angle 180 degrees; repetition time 1890 ms; echo time 13.8, 27.6, 41.4, 55.2, 69.0 ms; field of view 16 cm; 256 × 256 matrix Reconstructed Voxel 0.63 × 0.63 × 3 mm; 1 excitation; slice thickness 3 mm
Adelaide	3 T whole-body MR unit (Siemens Magnetom Skyra 3Tesla), using a dedicated 15 channel knee coil	T2 weighted fat-saturated 3D SPACE; repetition time 2200 msec; echo time 121 msec; field of view 16 cm; 320 × 320 matrix; 1 excitation; slice thickness 0.6 mm	T1-weighted Water Excitation 3D gradient-recalled acquisition VIBE; flip angle 16 degrees; repetition time 10.4 ms; echo time 5.7 ms; field of view 160 cm; 320 × 298 matrix; 1 excitation; slice thickness 1 mm	T2 mapping (multi echo acquisition); flip angle 180 degrees; repetition time 1750 ms; echo time 13.8/27.6/41.4/55.2/69 ms; field of view 16 cm; 320 × 320 matrix; 1 excitation; slice thickness 3 mm
Melbourne	3 T whole-body MR unit (Siemens, (Skyra 3 T) Magnetom Avanto Fit, Erlangen, Germany), using a dedicated Transmit/Receive 15 channel knee coil	T2 weighted fat-saturated 3D fast spin echo sequence; flip angle mode T2; repetition time 1200 ms; echo time 50 ms; field of view 16 cm; 230 × 320 matrix without interpolation not on; Recon Voxel 0.5 × 0.5 × 0.6 mm; 1 excitation; slice thickness 0.6 mm	T1-weighted fat-saturated 3D gradient-recalled acquisition; flip angle 30 degrees; repetition time 11.7 ms; echo time 5.61 ms; field of view 16 cm; 320 × 320 matrix Reconstructed Voxel 0.5 × 0.5 × 1.5 mm; 1 excitation; slice thickness 1.5 mm	T2 mapping (multi echo SE); flip angle 180 degrees; repetition time 1840 ms; echo time 11.7, 23.4, 35.1, 46.8, 58.5 ms; field of view 16 cm; 307 × 384 matrix Reconstructed Voxel 0.4 × 0.4 × 3 mm; 1 excitation; slice thickness 3 mm
Sydney	3 T whole-body MR unit (Siemens, Magnetom Skyra, Erlangen, Germany), using a dedicated Transmit/Receive 15 channel knee coil	T2 weighted fat-saturated 3D fast spin echo sequence; flip angle mode T2; repetition time 1000 ms; echo time 108 ms; field of view 16 cm; 256 × 256 matrix without interpolation Recon Voxel 0.6 × 0.6 × 0.6 mm; 1 excitation; slice thickness 0.6 mm	T1-weighted fat-saturated 3D gradient-recalled acquisition; flip angle 25 degrees; repetition time 11.6 ms; echo time 4.7 ms; field of view 16 cm; 256 × 256 matrix Reconstructed Voxel 0.6 × 0.6 × 1 mm; 1 excitation; slice thickness 1 mm	T2 mapping (multi echo SE); flip angle 180 degrees; repetition time 2150 ms; echo time 13.8, 27.6, 41.4, 55.2, 69.0 ms; field of view 16 cm; 384 × 326 matrix Reconstructed Voxel 0.42 × 0.42 × 3 mm; 1 excitation; slice thickness 3 mm

##### Knee effusion-synovitis

**Fig. 1:**
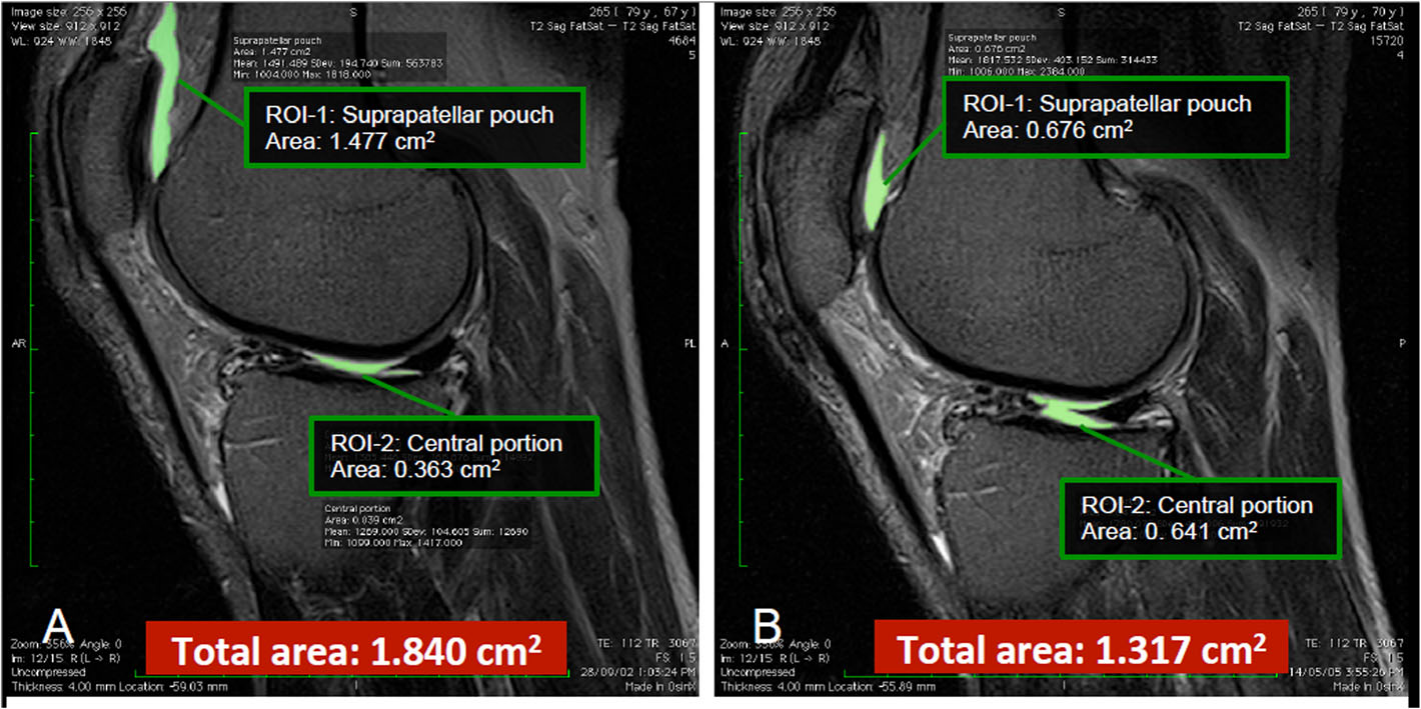
Example of changes in effusion-synovitis area (cm^2^). Effusion-synovitis size decreased from baseline to follow-up 2.6 years later (**a**, **b**). Effusion/synovitis are present in both the suprapatellar pouch and central portion. Overall, effusion size is smaller at follow-up MRI

Effusion-synovitis is defined as the presence of intra-articular fluid-equivalent signal on T2 weighted MRI sequence (Fig. 1). A modified Whole-Organ Magnetic Resonance Imaging Score (WORMS) scoring system will be utilised to assess effusion-synovitis (grade 0 to 3) semi-quantitatively in four regions of interest (ROI; suprapatellar pouch, central portion, posterior femoral recess, subpopliteal recess) in terms of the estimated maximal distention of the synovial cavity. The greatest score for any of the four ROI will be used as maximal effusion of the knee. The intraclass reliability assessed as weighted κ in 50 randomly selected images was 0.63–0.75 in different subregions, and the interclass inter-rater reliability was 0.65–0.79 [45].

Effusion-synovitis volume will be measured using a semi-automated segmentation method according to the intra-articular fluid-equivalent signal on a section-by-section basis in each ROI. The final 3-D volume rendering will be generated using commercial in-house imaging software. Change in effusion-synovitis volume is calculated by subtracting baseline values from follow-up values. The intra-rater reliability was 0.97 in the whole joint (0.98 in suprapatellar pouch and 0.95 in central portion). The inter-rater reliability was 0.99 in the whole joint (0.99 in suprapatellar pouch and 0.93 in central portion). Effusion-synovitis volume was highly correlated with effusion-synovitis score (rho 1/4 0.77, *P* < 0.01 for total; rho 1/4 0.91, *P* < 0.01 for suprapatellar pouch; and rho 1/4 0.77, *P* < 0.01 for central portion) [46].

Differentiating effusions from synovitis on MR images requires administration of a contrast agent (gadolinium), which may cause nephrotoxicity. While this is rare, we do not consider the benefits of such differentiation between synovitis and effusion justifies the risk to our study participants, and hence we have not used it.

##### Bone marrow lesions

Bone marrow lesions (BMLs) will be defined as an ill-defined hyperintensity area in the subchondral bone on MRI. BMLs will be assessed on the sagittal T2 weighted sequences at the medial tibial, medial femoral, lateral tibial, lateral femoral and patella sites using a modified WORMS scoring system [47]. The maximum size of each lesion will also be measured using software cursors applied to the greatest area of the lesion, as previously described [48]. We have demonstrated an intraclass correlation coefficient (ICC) of 0.97 [49] using this method. Total BML size will be calculated as the sum of every lesion within the medial tibial, medial femoral, lateral tibial, lateral femoral and patella sites.

#### Ultrasound

Knees will be imaged using an Esaote Mylab 70 VXG ultrasound machine in Perth (Table 4), with scans undertaken by a single sonographer. Images will be stored to be re-read to determine intra-reader reliability).

**Table 4:** Ultrasound parameters

	Suprapatellar pouch	Medial para patella	Lateral para patella
Knee position	30 degrees flexion	Neutral	Neutral
Probe plane	Longitudinal	Transverse	Transverse
Probe position	Probe on the distal thigh, at midline, longitudinal to its major axis	One end of the probe over the patella and the other end over the medial femoral condyle	One end of the probe over the patella and the other end over the lateral femoral condyle
Modes	GS and PD	GS and PD	GS and PD
Settings	To be optimised once machines known	To be optimised once machines known	To be optimised once machines known
Probe sweep	Move lateral to medial side	Proximal to distal side	Move proximal to distal side
Lesions to score	Synovitis (0–3) Synovial hypertrophy (0–1) Effusion (0–1) Effusion depth (mm) Synovial power Doppler signal (0–1)	Synovitis (0–3) Synovial hypertrophy (0–1) Effusion (0–1) Effusion depth (mm) Synovial power Doppler signal (0–1)	Synovitis (0–3) Synovial hypertrophy (0–1) Effusion (0–1) Effusion depth (mm) Synovial power Doppler signal (0–1)
Images to store	Midline longitudinal with the effusion depth measurement in situ	Representative image	Representative image

*GS* grey scale, *PD* power Doppler

Ultrasounds completed in Perth only

Synovitis in the supra patella pouch will be defined as a combination of synovial hypertrophy and effusion (using Outcome Measures in Rheumatology (OMERACT) definitions [50]) and scored on a 0–3 scale with grade 0 = no synovitis, grade 1 = minimal distension of the recess by abnormal internal hypoechoic or anechoic (relative to subdermal fat tissue) material, grade 2 = moderate distension or enlargement of the recess by abnormal internal hypoechoic or anechoic (relative to subdermal fat tissue) material with flat or concave superficial limit, and grade 3 = severe distension or enlargement of the recess by abnormal internal hypoechoic or anechoic (relative to subdermal fat tissue) material with bulging superficial limit. Depth of effusion (mm) will be measured only in the suprapatellar pouch (SPP), from a still midline longitudinal image, measuring the maximal effusion depth in this plane.

In the SPP, medial parapatellar region and lateral parapatellar region, synovial hypertrophy, effusion and power doppler signal (defined using OMERACT definitions [50]) will be scored as absent (0) or present (1).

#### Pressure pain threshold testing

Pressure pain threshold (PPT) testing will be conducted using a pressure algometer (FDN200, Wagner Instruments, USA) with a probe area of 1 cm^2^, which exerts pressures of up to 200 N/cm^2^/2000 kPa. The PPT is determined using an ascending stimulus intensity (slowly increasing ramps of 50 kPa/s (~ 0.5 kg/cm^2^s) applied bilaterally over the three test sites: the affected joint (knee, assessed over the tibial below the medial joint line), an unaffected joint (ipsilateral proximal radioulnar joint), and over the thenar eminence. The study participant is asked to notify the examiner as soon as the sensation becomes painful, and the pressure exerted recorded. The PPT is determined by the mean of the threshold from a series of three stimulus intensities each applied as a slowly increasing ramp of 50 kPa/s [51].

#### OMERACT–OARSI responder criteria

Response to krill oil will be assessed using the OMERACT–Osteoarthritis Society International (OARSI) set of response criteria [41]. Participants will be classed as responding if they have high improvement in pain (using the VAS) or function (using WOMAC function scale) of ≥ 50% and absolute change ≥ 20; or if they have positive relative (≥ 20%) or absolute (≥ 10) change in two out of three of pain, function or patient’s global assessment.

#### Lower limb muscle strength

Lower limb muscle strength is a key correlate of pain and typically increases when pain reduces [52]. We will assess lower limb leg strength in both legs simultaneously, using a dynamometer (TTM Muscular Meter, Tokyo, Japan) as previously described [21]. This is done in duplicate and the mean of the two estimates is used. The muscles measured in this technique are mainly the quadriceps and hip flexors. The previously published repeatability estimate (Cronbach’s α) for this method is 0.91 [53].

#### Measure of intermittent and constant osteoarthritis pain

Intermittent and constant osteoarthritis pain (ICOAP) is a multidimensional OA-specific measure designed to comprehensively evaluate the pain experience in people with hip or knee OA. This includes pain intensity, frequency, and impact on mood, sleep and quality of life, independent of the effect of pain on physical function [54], over the past 7 days. It contains 11 items in two domains with two supplementary items on intermittent pain predictability, and uses rating scales with five categories of response, e.g., “not at all,” “mildly,” “moderately,” “severely,” and “extremely” [54]. Each ICOAP item is scored from 0 to 4; missing data are dealt with according to rules in the user guide [55]. Scores are produced for each subscale separately by summing subscale scores for each item and then normalising each score from 0 (no pain) to 100 (extreme pain). The ICOAP is reliable (Cronbach’s α 0.93, ICC 0.85 [54]) and valid [54].

#### Quality of life

Quality of life will be assessed using the Assessment of Quality of Life (AQoL-6D) questionnaire, which assesses six separately scored dimensions (Independent Living, Relationships, Mental Health, Coping, Pain and Senses), each with variable item numbers and response levels [56]. The AQoL-6D has good psychometric properties [57]. Utility scores will be calculated based on methods published on the AQoL website [56] with a range 0–1 where 0 indicates the worst health state and 1 the best.

#### Blood samples

Fasting blood samples will be obtained from study participants at screening, 12 and 24 weeks. Samples will be analysed for fasting glucose, lipids (total, HDL and LDL) and hsCRP. The blood will be either tested fresh or stored at − 80 °C.

### Other assessments

#### Radiographs

A standing anteroposterior semiflexed radiograph of the ‘study’ knee will be performed at screening. X-rays will be scored for joint space narrowing and osteophytes on a four point scale (0–3) using the OARSI atlas [39]. In our hands this method has very high reproducibility with an ICC of 0.98 for joint space narrowing and 0.99 for osteophytes [58].

#### Anthropometry

These include height (stadiometer) and weight (electronic scales) and body mass index (BMI) (weight/height^2^) measured at weeks 0, 12 and 24.

#### painDETECT

Neuropathic pain will be assessed by the painDETECT questionnaire (− 1 to 38) at screening to provide information on level of nociceptive and/or neuropathic contributions to pain. A painDETECT score < 12 is defined as unlikely neuropathic pain, and 13–18 as possible neuropathic pain [59].

#### Concomitant medication

Use of pain medicines will be recorded by questionnaire at all visits. Participants will be asked to keep medications as stable as possible (including NSAIDs) and use paracetamol as rescue medication. Use of fish and krill oil during the trial will not be permitted, and use must cease 2 weeks prior to randomisation. Participants who commence anti-coagulant therapy will be withdrawn from the trial. We will assess analgesic use from the medication data.

#### Treatment guessing and adherence

Study participants will be asked what treatment they think they received at the 12- and 24-week assessments with the following options: krill oil, placebo, or not sure.

Adherence to treatment will also be assessed at the 12- and 24-week assessments by standard pill count methods [60].

#### Safety assessment

Adverse events will be monitored throughout the study. Standard safety and efficacy monitoring will be performed through regular face-to-face visits and phone calls between visits. The patients are requested to report any adverse events to the research staff spontaneously. Details of the adverse event and its relationship with study intervention will be recorded and reported to the local Human Research Ethics Committees in accordance with the requirements of individual committees.

### Sample size calculations

Using data from another trial conducted in our centre [52], assuming 10-mm difference between krill oil and placebo on the VAS pain scale (reduction in VAS pain scores in the placebo group by − 15.5 ± 25.5 mm over 12 weeks) and using assumptions of 90% power and 5% probability of type 1 error (alpha = 0.05), we will need 234 participants. Adjusting for 10% loss to follow-up, we need 260 participants (130 in each arm). Based on data on effusion-synovitis volume in our vitamin D RCT for knee OA, there was 13.7 ml (SD = 10.7) in the placebo group and 13.6 ml (8.1) in the active group (in those with effusion-synovitis at enrolment, i.e., 60%) at baseline [61]. With 234 subjects we will have 90% power to detect a difference of 4.5 ml. It is likely we will see at least a reduction of this amount with a treatment that effectively targets inflammation. What level reflects clinical significance is uncertain but this level is approximately 2.5 times the change that could be expected with measurement error. Furthermore, modelled data from our long-term Tasmanian cohort demonstrates that a reduction in effusion-synovitis size by 4.5 ml will decrease the need for joint replacement by 30% over 13 years (unpublished data).

### Statistical analysis

The primary analyses will be intention-to-treat analyses of primary and secondary outcomes. Per protocol analyses will be performed as the secondary analyses.

Changes in knee pain, knee effusion-synovitis size and any other outcomes which were collected using a linear scale will be analysed using a linear mixed model with treatment, month and their interaction (treatment × month) as covariates, as well as outcome measured at baseline and the baseline interaction with month. Incidence of adverse events will be assessed using log binomial regression. Correlated data within trial centres and the repeated measures will be addressed using trial centre and patient identification as random intercepts. Month will be treated as random effect to allow different treatment effects among patients over time. The sensitivity of models to the structure for the random effects and covariance structures will be assessed using likelihood ratio tests. Change in outcome measures within each group and differences in changes between groups from baseline to follow-up will be calculated using linear combinations of the estimated coefficients. If there are baseline imbalances in covariates between treatment groups, we will consider adjusting for them based on whether we regard the imbalance as clinically significant. Missing data caused by loss to follow-up and non-responses will be addressed by adding variables that have complete data at baseline and can explain missingness to the regression models.

Secondary analysis for missing data will be performed in people with full medication adherence (e.g., > 80% of softgels) and using imputation. Baseline variables with complete data will be used for data imputation assuming missing at random.

Subgroup analyses will be performed to examine which subgroups may respond better to treatment. These will include effusion size, radiographic knee OA, co-pathology present on MRI, pain characteristics, levels of inflammatory markers and serum lipid measures. Statistical significance will be set as a two-sided *P* value < 0.05.

### Data integrity and management

Data will be recorded using case report forms and processed centrally at the Menzies Institute for Medical Research, University of Tasmania. The hard copies of case report forms will be stored in a locked area at each study site with secured and restricted access. The electronic data will be stored on password-protected servers with restricted access. All data collected will be kept strictly confidential. Daily backups of all electronic data will occur to minimise any risk of lost data. Data transfer will be encrypted with all data de-identified. Only members of the research team who need to contact study patients, enter data or perform data quality control will have access to patient information.

After study completion, paper copies of data will be archived in secure storage. Identifiers will not be removed in case follow-up of study patients is necessary; however, electronic data will continue to be kept in a secure electronic database. This will remain password protected and with access given only to the study investigators unless otherwise authorised by the study team.

### Withdrawal

If patients withdraw from the study before 6 months of follow-up, the reason and date will be recorded. An early MRI will be considered for participants who can not make the final visit.

### Roles and responsibilities and monitoring

The University of Tasmania (as the trial sponsor) and the principal investigators are responsible for all aspects of the trial, including design, conduct and oversight. The principal investigators will monitor the conduct and progress of the project at each site. The trial coordinator will visit each study site to make sure that all trial procedures are compliant with the trial protocol. The principal investigators and the research team will have regular teleconferences to ensure efficient study execution and ongoing monitoring of the study progress, with summary documents circulated after each meeting. Krill oil has a good safety profile [31] and is available over-the-counter, so we do not plan to use a data safety monitoring board. The trial is also being monitored at each site by a practicing rheumatologist.

### Dissemination plans

The results of this study will be presented at conferences and published in scientific journals. Any notes or publications arising from our research will be de-identified. Only aggregate statistical results will be presented.

The outcomes of the project will be disseminated to study patients using non-technical language. Dissemination of the overall study findings to the patients will occur in a de-identified manner and be based on the entire study population. The scientific paper will be available for dissemination to study participants.

## Discussion

We propose a multicentre, randomised, double blind placebo-controlled trial to determine whether krill oil 2 g/day improves knee pain and reduces size of knee effusion-synovitis compared to placebo in people with clinical knee OA, significant knee pain and knee effusion-synovitis. If krill oil proves effective, it will offer a novel therapeutic approach to reduce or slow progression of knee OA.

Krill oil is theorised to have an effect via both anti-inflammatory and anti-oxidative mechanisms. Oxidative stress and inflammation have important roles in OA pathogenesis and krill oil therapy may be beneficial in treating OA. Krill oil (300 mg/day) reduced inflammation (as measured by CRP) by 30% in 30 days vs 25% increase in patients receiving placebo [34]. This may have important clinical implications, with low level inflammation associated with increased loss of tibial cartilage volume (β = − 1.18% per annum per quartile of IL-6) [62]). Accordingly, trials are starting to use MRI-assessed effusion/synovitis as a treatment target. In a trial using low dose oral prednisolone for painful hand OA, MRI-assessed effusion-synovitis was associated with pain cross-sectionally, but not hand pain as assessed by VAS; however, effusions did not change over time with treatment or predict response to treatment [63].

Effusion and synovitis can also be assessed using ultrasound; such effusions predict knee replacements independent of severity of radiographic damage and pain [20]. Presence of ultrasound-detected knee effusions has been used to select people who might respond well to oral methotrexate, which demonstrated efficacy in an open-label trial, albeit without a control group [64]. However, the reproducibility of ultrasound is largely dependent on the operator and ultrasound is not yet well utilised in clinical trials of knee OA. This study will enable comparisons of MRI vs ultrasound indices in assessing change in effusion volume and provide evidence of which imaging modality is of most use in this context.

In summary, knee OA is a major but poorly understood public health problem with no pharmacologic therapies that affect disease progression. Two small trials [34, 35] suggest that krill oil will improve knee pain and may reduce systemic inflammation in people with OA. If krill oil can reduce knee pain and effusion size in knee OA, study findings will be readily translated into clinical practice as krill oil is already popular and available over the counter despite limited evidence of efficacy.

### Trial status

Trial status: Completed recruiting.

Protocol version number and date: Scientific protocol V2 (08 April 2016), Standard operating procedures: V4 (Hobart site, 13 November 2017), V3 (Adelaide site, 23 August 2016).

Date recruitment began: 06 December 2016, recruitment complete July 2019.

## Supplementary information

**Additional file 1.** SPIRIT 2013 checklist: Recommended items to address in a clinical trial protocol and related documents.

## Data Availability

Data generated from this study will not be deposited in a public repository due to privacy and consent restrictions. De-identified data can be made available from the corresponding author on reasonable request, subject to a data sharing agreement.

## Abbreviations

3-D: Three-dimensional
ACR: American College of Rheumatology
AE: Adverse event
AQoL: The Assessment of Quality of Life
BMI: Body mass index
BMLs: Bone marrow lesions
CONSORT: Consolidated Standards of Reporting Trials
DALYs: Disability-adjusted life years
DHA: Decosahexanoic acid
DMOADs: Disease-modifying OA drugs
EPA: Eicosapentanoic acids
GS: Grey scale
HDL: High density lipoproteins
hsCRP: High sensitive C reactive protein
ICC: Intraclass correlation coefficient
ICOAP: Measure of intermittent and constant osteoarthritis pain
IL: Interleukin
JSN: Joint space narrowing
KARAOKE: Randomised trial of krill oil for osteoarthritis of the knee
LDL: Low density lipoproteins
MRI: Magnetic resonance imaging
NHMRC: National Health and Medical Research Council
NSAIDs: Non-steroidal anti-inflammatory drug
OA: Osteoarthritis
OARSI: Osteoarthritis Research Society International
OMERACT: Outcome Measures in Rheumatology
PD: Power Doppler
PPT: Pressure pain testing
QoL: Quality of life
RA: Rheumatoid arthritis
RCTs: Randomised controlled trial
ROI: Regions of interest
SD: Standard deviation
TASOAC: Tasmanian Older Adult Cohort
TNF-α: Tumour necrosis factor-α
UTAS: University of Tasmania
UTN: Universal Trial Number
VAS: Visual analogue scale
WOMAC: Western Ontario and McMasters Universities Osteoarthritis Index

## References

[1] Murray CJ, Vos T, Lozano R, Naghavi M, Flaxman AD, Michaud C, Ezzati M, Shibuya K, Salomon JA, Abdalla S (2012). Disability-adjusted life years (DALYs) for 291 diseases and injuries in 21 regions, 1990-2010: a systematic analysis for the Global Burden of Disease Study 2010. Lancet.

[2] Cross M, Smith E, Hoy D, Nolte S, Ackerman I, Fransen M, Bridgett L, Williams S, Guillemin F, Hill CL (2014). The global burden of hip and knee osteoarthritis: estimates from the global burden of disease 2010 study. Ann Rheum Dis.

[3] Tallon D, Chard J, Dieppe P (2000). Exploring the priorities of patients with osteoarthritis of the knee. Arthritis Care Res.

[4] Conaghan PG, Peloso PM, Everett SV, Rajagopalan S, Black CM, Mavros P, Arden NK, Phillips CJ, Rannou F, van de Laar MA (2015). Inadequate pain relief and large functional loss among patients with knee osteoarthritis: evidence from a prospective multinational longitudinal study of osteoarthritis real-world therapies. Rheumatology (Oxford).

[5] Lane NE, Brandt K, Hawker G, Peeva E, Schreyer E, Tsuji W, Hochberg MC (2011). OARSI-FDA initiative: defining the disease state of osteoarthritis. Osteoarthr Cartil.

[6] Jones G (2013). Sources of pain in osteoarthritis: Implications for therapy. Int J Clin Rheumatol.

[7] Spector TD, Hart DJ, Nandra D, Doyle DV, Mackillop N, Gallimore JR, Pepys MB (1997). Low-level increases in serum C-reactive protein are present in early osteoarthritis of the knee and predict progressive disease. Arthritis Rheum.

[8] Scanzello CR, Goldring SR (2012). The role of synovitis in osteoarthritis pathogenesis. Bone.

[9] Deveza LA, Melo L, Yamato TP, Mills K, Ravi V, Hunter DJ (2017). Knee osteoarthritis phenotypes and their relevance for outcomes: a systematic review. Osteoarthr Cartil.

[10] Jin X, Beguerie JR, Zhang W, Blizzard L, Otahal P, Jones G, Ding C (2015). Circulating C reactive protein in osteoarthritis: a systematic review and meta-analysis. Ann Rheum Dis.

[11] Pearle AD, Scanzello CR, George S, Mandl LA, DiCarlo EF, Peterson M, Sculco TP, Crow MK (2007). Elevated high-sensitivity C-reactive protein levels are associated with local inflammatory findings in patients with osteoarthritis. Osteoarthr Cartil.

[12] Stannus OP, Jones G, Blizzard L, Cicuttini FM, Ding C (2013). Associations between serum levels of inflammatory markers and change in knee pain over 5 years in older adults: a prospective cohort study. Ann Rheum Dis.

[13] Hanna FS, Bell RJ, Cicuttini FM, Davison SL, Wluka AE, Davis SR (2008). High sensitivity C-reactive protein is associated with lower tibial cartilage volume but not lower patella cartilage volume in healthy women at mid-life. Arthritis Res Ther.

[14] Sharif M, Shepstone L, Elson CJ, Dieppe PA, Kirwan JR (2000). Increased serum C reactive protein may reflect events that precede radiographic progression in osteoarthritis of the knee. Ann Rheum Dis.

[15] Yusuf E, Kortekaas MC, Watt I, Huizinga TW, Kloppenburg M (2011). Do knee abnormalities visualised on MRI explain knee pain in knee osteoarthritis? A systematic review. Ann Rheum Dis.

[16] Hill CL, Hunter DJ, Niu J, Clancy M, Guermazi A, Genant H, Gale D, Grainger A, Conaghan P, Felson DT (2007). Synovitis detected on magnetic resonance imaging and its relation to pain and cartilage loss in knee osteoarthritis. Ann Rheum Dis.

[17] Zhang Y, Nevitt M, Niu J, Lewis C, Torner J, Guermazi A, Roemer F, McCulloch C, Felson DT (2011). Fluctuation of knee pain and changes in bone marrow lesions, effusions, and synovitis on magnetic resonance imaging. Arthritis Rheum.

[18] Wang X, Jin X, Han W, Cao Y, Halliday A, Blizzard L, Pan F, Antony B, Cicuttini F, Jones G, Ding C (2016). Cross-sectional and longitudinal associations between knee joint effusion synovitis and knee pain in older adults. J Rheumatol.

[19] Wang Xia, Blizzard Leigh, Halliday Andrew, Han Weiyu, Jin Xingzhong, Cicuttini Flavia, Jones Graeme, Ding Changhai (2014). Association between MRI-detected knee joint regional effusion-synovitis and structural changes in older adults: a cohort study. Annals of the Rheumatic Diseases.

[20] Conaghan PG, D'Agostino MA, Le Bars M, Baron G, Schmidely N, Wakefield R, Ravaud P, Grassi W, Martin-Mola E, So A (2010). Clinical and ultrasonographic predictors of joint replacement for knee osteoarthritis: results from a large, 3-year, prospective EULAR study. Ann Rheum Dis.

[21] Abou-Raya A, Abou-Raya S, Khadrawi T, Helmii M (2014). Effect of low-dose oral prednisolone on symptoms and systemic inflammation in older adults with moderate to severe knee osteoarthritis: a randomized placebo-controlled trial. J Rheumatol.

[22] Aitken D, Laslett LL, Pan F, Haugen IK, Otahal P, Bellamy N, Bird P, Jones G (2018). A randomised double-blind placebo-controlled crossover trial of HUMira (adalimumab) for erosive hand OsteoaRthritis - the HUMOR trial. Osteoarthr Cartil.

[23] Goldberg RJ, Katz J (2007). A meta-analysis of the analgesic effects of omega-3 polyunsaturated fatty acid supplementation for inflammatory joint pain. Pain.

[24] Fortin PR, Lew RA, Liang MH, Wright EA, Beckett LA, Chalmers TC, Sperling RI (1995). Validation of a meta-analysis: the effects of fish oil in rheumatoid arthritis. J Clin Epidemiol.

[25] Lee YH, Bae SC, Song GG (2012). Omega-3 polyunsaturated fatty acids and the treatment of rheumatoid arthritis: a meta-analysis. Arch Med Res.

[26] Proudman SM, James MJ, Spargo LD, Metcalf RG, Sullivan TR, Rischmueller M, Flabouris K, Wechalekar MD, Lee AT, Cleland LG (2015). Fish oil in recent onset rheumatoid arthritis: a randomised, double-blind controlled trial within algorithm-based drug use. Ann Rheum Dis.

[27] Baker KR, Matthan NR, Lichtenstein AH, Niu J, Guermazi A, Roemer F, Grainger A, Nevitt MC, Clancy M, Lewis CE (2012). Association of plasma n-6 and n-3 polyunsaturated fatty acids with synovitis in the knee: the MOST study. Osteoarthr Cartil.

[28] Senftleber Ninna, Nielsen Sabrina, Andersen Jens, Bliddal Henning, Tarp Simon, Lauritzen Lotte, Furst Daniel, Suarez-Almazor Maria, Lyddiatt Anne, Christensen Robin (2017). Marine Oil Supplements for Arthritis Pain: A Systematic Review and Meta-Analysis of Randomized Trials. Nutrients.

[29] Hill CL, March LM, Aitken D, Lester SE, Battersby R, Hynes K, Fedorova T, Proudman SM, James M, Cleland LG, Jones G (2016). Fish oil in knee osteoarthritis: a randomised clinical trial of low dose versus high dose. Ann Rheum Dis.

[30] Tou JC, Jaczynski J, Chen YC (2007). Krill for human consumption: nutritional value and potential health benefits. Nutr Rev.

[31] Ulven SM, Kirkhus B, Lamglait A, Basu S, Elind E, Haider T, Berge K, Vik H, Pedersen JI (2011). Metabolic effects of krill oil are essentially similar to those of fish oil but at lower dose of EPA and DHA, in healthy volunteers. Lipids.

[32] Ierna M, Kerr A, Scales H, Berge K, Griinari M (2010). Supplementation of diet with krill oil protects against experimental rheumatoid arthritis. BMC Musculoskelet Disord.

[33] Vigerust NF, Bjorndal B, Bohov P, Brattelid T, Svardal A, Berge RK (2013). Krill oil versus fish oil in modulation of inflammation and lipid metabolism in mice transgenic for TNF-alpha. Eur J Nutr.

[34] Deutsch L (2007). Evaluation of the effect of Neptune Krill Oil on chronic inflammation and arthritic symptoms. J Am Coll Nutr.

[35] Suzuki Y, Fukushima M, Sakuraba K, Sawaki K, Sekigawa K (2016). Krill oil improves mild knee joint pain: A randomized control trial. PLoS One.

[36] Schulz KF, Altman DG, Moher D (2010). CONSORT 2010 statement: updated guidelines for reporting parallel group randomized trials. Ann Intern Med.

[37] Altman R, Asch E, Bloch D, Bole G, Borenstein D, Brandt K, Christy W, Cooke TD, Greenwald R, Hochberg M (1986). Development of criteria for the classification and reporting of osteoarthritis. Classification of osteoarthritis of the knee. Diagnostic and Therapeutic Criteria Committee of the American Rheumatism Association. Arthritis Rheum.

[38] Wang X, Jin X, Blizzard L, Antony B, Han W, Zhu Z, Cicuttini F, Wluka AE, Winzenberg T, Jones G, Ding C (2017). Associations between knee effusion-synovitis and joint structural changes in patients with knee osteoarthritis. J Rheumatol.

[39] Altman RD, Hochberg M, Murphy WA, Wolfe F, Lequesne M (1995). Atlas of individual radiographic features in osteoarthritis. Osteoarthr Cartil.

[40] Taves DR (1974). Minimization: a new method of assigning patients to treatment and control groups. Clin Pharmacol Ther.

[41] Pham T, van der Heijde D, Altman RD, Anderson JJ, Bellamy N, Hochberg M, Simon L, Strand V, Woodworth T, Dougados M (2004). OMERACT-OARSI initiative: Osteoarthritis Research Society International set of responder criteria for osteoarthritis clinical trials revisited. Osteoarthr Cartil.

[42] Bellamy N, Buchanan WW, Goldsmith CH, Campbell J, Stitt LW (1988). Validation study of WOMAC: a health status instrument for measuring clinically important patient relevant outcomes to antirheumatic drug therapy in patients with osteoarthritis of the hip or knee. J Rheumatol.

[43] Kersten P, White PJ (2010). Tennant A: The visual analogue WOMAC 3.0 scale--internal validity and responsiveness of the VAS version. BMC Musculoskelet Disord.

[44] Bellamy N. WOMAC Osteoarthritis Index User Guide. Version VII. Brisbane, Australia; 2005. http://www.womac.org/womac/womac_userguide.htm

[45] Wang X, Blizzard L, Halliday A, Han W, Jin X, Cicuttini F, Jones G, Ding C (2016). Association between MRI-detected knee joint regional effusion-synovitis and structural changes in older adults: a cohort study. Ann Rheum Dis.

[46] Wang X, Cicuttini F, Jin X, Wluka AE, Han W, Zhu Z, Blizzard L, Antony B, Winzenberg T, Jones G, Ding C (2017). Knee effusion-synovitis volume measurement and effects of vitamin D supplementation in patients with knee osteoarthritis. Osteoarthr Cartil.

[47] Doré D, Martens A, Quinn S, Ding C, Winzenberg T, Zhai G, Pelletier JP, Martel-Pelletier J, Abram F, Cicuttini F, Jones G (2010). Bone marrow lesions predict site-specific cartilage defect development and volume loss: a prospective study in older adults. Arthritis Res Ther.

[48] Laslett LL, Doré DA, Quinn SJ, Boon P, Ryan E, Winzenberg TM, Jones G (2012). Zoledronic acid reduces knee pain and bone marrow lesions over 1 year: a randomised controlled trial. Ann Rheum Dis.

[49] Doré D, Quinn S, Ding C, Winzenberg T, Zhai G, Cicuttini F, Jones G (2010). Natural history and clinical significance of MRI-detected bone marrow lesions at the knee: a prospective study in community dwelling older adults. Arthritis Res Ther.

[50] Wakefield RJ, Balint PV, Szkudlarek M, Filippucci E, Backhaus M, D'Agostino MA, Sanchez EN, Iagnocco A, Schmidt WA, Bruyn GA (2005). Musculoskeletal ultrasound including definitions for ultrasonographic pathology. J Rheumatol.

[51] Rolke R, Magerl W, Campbell KA, Schalber C, Caspari S, Birklein F, Treede RD (2006). Quantitative sensory testing: a comprehensive protocol for clinical trials. Eur J Pain.

[52] Laslett LL, Quinn SJ, Darian-Smith E, Kwok M, Fedorova T, Korner H, Steels E, March L, Jones G (2012). Treatment with 4Jointz reduces knee pain over 12 weeks of treatment in patients with clinical knee osteoarthritis: a randomised controlled trial. Osteoarthr Cartil.

[53] Jones G, Glisson M, Hynes K, Cicuttini F (2000). Sex and site differences in cartilage development: a possible explanation for variations in knee osteoarthritis in later life. Arthritis Rheum.

[54] Hawker GA, Davis AM, French MR, Cibere J, Jordan JM, March L, Suarez-Almazor M, Katz JN, Dieppe P (2008). Development and preliminary psychometric testing of a new OA pain measure--an OARSI/OMERACT initiative. Osteoarthr Cartil.

[55] Measure of Intermittent and Constant Osteoarthritis Pain: ICOAP User’s Guide [https://www.oarsi.org/sites/default/files/docs/2013/icoap_users_guide_07072010.pdf]. Accessed 13 Nov 2018.

[56] AQoL-6D [http://www.aqol.com.au/aqolquestionnaires/56.html]. Accessed 13 Nov 2018.

[57] Richardson JR, Peacock SJ, Hawthorne G, Iezzi A, Elsworth G, Day NA (2012). Construction of the descriptive system for the Assessment of Quality of Life AQoL-6D utility instrument. Health Qual Life Outcomes.

[58] Jones G, Ding C, Scott F, Glisson M, Cicuttini F (2004). Early radiographic osteoarthritis is associated with substantial changes in cartilage volume and tibial bone surface area in both males and females. Osteoarthr Cartil.

[59] Freynhagen R, Baron R, Gockel U, Tolle TR (2006). painDETECT: a new screening questionnaire to identify neuropathic components in patients with back pain. Curr Med Res Opin.

[60] Lee JK, Grace KA, Foster TG, Crawley MJ, Erowele GI, Sun HJ, Turner PT, Sullenberger LE, Taylor AJ (2007). How should we measure medication adherence in clinical trials and practice?. Ther Clin Risk Manag.

[61] Jin X, Jones G, Cicuttini F, Wluka A, Zhu Z, Han W, Antony B, Wang X, Winzenberg T, Blizzard L, Ding C (2016). Effect of vitamin D supplementation on tibial cartilage volume and knee pain among patients with symptomatic knee osteoarthritis: A randomized clinical trial. JAMA.

[62] Stannus O, Jones G, Cicuttini F, Parameswaran V, Quinn S, Burgess J, Ding C (2010). Circulating levels of IL-6 and TNF-alpha are associated with knee radiographic osteoarthritis and knee cartilage loss in older adults. Osteoarthr Cartil.

[63] Wenham CY, Hensor EM, Grainger AJ, Hodgson R, Balamoody S, Dore CJ, Emery P, Conaghan PG (2012). A randomized, double-blind, placebo-controlled trial of low-dose oral prednisolone for treating painful hand osteoarthritis. Rheumatology (Oxford).

[64] Wenham CY, Grainger AJ, Hensor EM, Caperon AR, Ash ZR, Conaghan PG (2013). Methotrexate for pain relief in knee osteoarthritis: an open-label study. Rheumatology (Oxford).

